# Association Mapping of Drought Tolerance Indices in Ethiopian Durum Wheat (*Triticum turgidum* ssp. durum)

**DOI:** 10.3389/fpls.2022.838088

**Published:** 2022-05-26

**Authors:** Kefyalew Negisho, Surafel Shibru, Andrea Matros, Klaus Pillen, Frank Ordon, Gwendolin Wehner

**Affiliations:** ^1^National Agricultural Biotechnology Research Center, Ethiopian Institute of Agricultural Research (EIAR), Holeta, Ethiopia; ^2^Melkassa Research Center, Ethiopian Institute of Agricultural Research (EIAR), Melkassa, Ethiopia; ^3^Julius Kühn Institute (JKI), Institute for Resistance Research and Stress Tolerance, Quedlinburg, Germany; ^4^Institute of Agricultural and Nutritional Sciences, Martin Luther University, Halle, Germany

**Keywords:** Ethiopia, durum wheat, drought tolerance indices, GWAS, QTLs, field studies

## Abstract

Ethiopia is a major producer of durum wheat in sub-Saharan Africa. However, its production is prone to drought stress as it is fully dependent on rain, which is erratic and unpredictable. This study aimed to detect marker-trait associations (MTAs) and quantitative trait loci (QTLs) related to indices. Six drought tolerance indices, i.e., drought susceptibility index (DSI), geometric mean productivity (GMP), relative drought index (RDI), stress tolerance index (STI), tolerance index (TOL), and yield stability index (YSI) were calculated from least-square means (lsmeans) of grain yield (GY) and traits significantly (*p* < 0.001) correlated with grain yield (GY) under field drought stress (FDS) and field non-stress (FNS) conditions. GY, days to grain filling (DGF), soil plant analysis development (SPAD) chlorophyll meter, seeds per spike (SPS), harvest index (HI), and thousand kernel weight (TKW) were used to calculate DSI, GMP, RDI, STI, TOL, and YSI drought indices. Accessions, DW084, DW082, DZ004, C037, and DW092 were selected as the top five drought-tolerant based on DSI, RDI, TOL, and YSI combined ranking. Similarly, C010, DW033, DW080, DW124-2, and C011 were selected as stable accessions based on GMP and STI combined ranking. A total of 184 MTAs were detected linked with drought indices at –log_10_p ≥ 4.0,79 of which were significant at a false discovery rate (FDR) of 5%. Based on the linkage disequilibrium (LD, *r*^2^ ≥ 0.2), six of the MTAs with a positive effect on GY-GMP were detected on chromosomes 2B, 3B, 4A, 5B, and 6B, explaining 14.72, 10.07, 26.61, 21.16, 21.91, and 22.21% of the phenotypic variance, respectively. The 184 MTAs were clustered into 102 QTLs. Chromosomes 1A, 2B, and 7A are QTL hotspots with 11 QTLs each. These chromosomes play a key role in drought tolerance and respective QTL may be exploited by marker-assisted selection for improving drought stress tolerance in wheat.

## Introduction

Globally, drought is a serious abiotic factor challenging crop production, productivity, and quality. It is enhanced by climate change leading to food and livelihood crises (Lobell et al., 2011). Singh et al. (2016) reported total crop failure and death of livestock due to drought in Ethiopia affecting nearly 10 million people, especially in the northern part of the country. Hence, Ethiopia is experiencing significant climate-induced drought and water-related stresses on crop and livestock productivity (Brown et al., 2017). Durum wheat (2*n* = 28, AABB, *Triticum turgidum* L. ssp. durum) is the most commonly cultivated form of allotetraploid wheat and is grown in 8% of the world's wheat area (FAO, 2016). In Ethiopia, durum wheat nearly accounts for 15–20% of wheat production and covers 30% of wheat cultivated land areas (Negassa et al., 2013). In Ethiopia, durum wheat is not only a staple crop for food security but also it becomes a major cash crop having 10–20% higher prices than bread wheat (Sall et al., 2019).

Ethiopia is one of the world's eight major Vavilovian centers of origin of crop plants, such as durum wheat and a major durum wheat producer, in sub-Sharan Africa (Vavilov, 1951; Sall et al., 2019). However, its production is fully dependent on rain, which is erratic and unpredictable, particularly in the low altitude regions (Simane et al., 1994). Ethiopia is currently harvesting crops only from 14 million out of 51.3 million hectares of arable lands [Tsegaye, 2017; Central Statistical Agency of Ethiopia (CSA)., 2018]. This is primarily due to drought stress and the lack of irrigation facilities among other production constraints. Therefore, the selection of drought-tolerant durum wheat genotypes has paramount importance in expanding its production to the untapped potential production areas and to use drought-tolerant genotypes in wheat improvement programs. The huge genetic diversity in Ethiopian durum wheat landraces could be a potential gene pool for national and international wheat improvements (Mengistu et al., 2015; Negisho et al., 2021). Thereby, the identification and use of drought-tolerant accessions from the existing genetic diversities could help to overcome the drastic effect of drought (Van Oosten et al., 2016).

Drought indices provide a mathematical measure for yield loss under drought stress conditions as compared to non-stress conditions in screening drought-tolerant genotypes (Fernandez, 1992; Mitra, 2001). They have been widely used for screening drought-tolerant genotypes in durum wheat (Patel et al., 2019), bread wheat (Abdolshahi et al., 2012; Song et al., 2017), barley (Sallam et al., 2019), and maize (Naghavi et al., 2013; Yousefi, 2015). The drought susceptibility index (DSI) is used to measure yield stability in wheat genotypes that apprehends the changes in both drought stress and non-stress environments (Fischer and Maurer, 1978). Guttieri et al. (2001) suggested genotypes with DSI values of <1 showing tolerance to drought stress. Genotypes with high values for yield stability index (YSI) (Bouslama and Schapaugh, 1984) and relative drought index (RDI) (Fischer et al., 1998) are generally regarded as stable under stress and non-stress conditions. Rosielle and Hamblin (1981) also proposed drought stress tolerance (TOL) criteria based on mean yield from drought stress and non-stress conditions. Similarly, the stress tolerance index (STI) (Fernandez, 1992) and geometric mean productivity (GMP) (Ramirez-Vallejo and Kelly, 1998) are useful indices for the identification of stable genotypes, which produce high yield under drought stress and higher or optimum yield under non-stress.

Quantitative trait loci have been detected for grain yield-related drought tolerance indices traits in wheat (Edae et al., 2014; Maccaferri et al., 2015; Qaseem et al., 2019) and chickpea (Kale et al., 2015). However, research on the identification of QTLs associated with drought tolerance indices for traits other than grain yield is scarce. For instance, Sukumaran et al. (2018) detected QTLs associated with drought indices (SSI, TOL, STI) calculated from grain yield (GY), thousand-grain weight (TGW), and grain number in durum wheat. Similarly, Ballesta et al. (2020) identified QTL-rich regions associated with drought indices (SSI, TOL, STI, and YSI) derived from grain yield (GY), TKW, and kernels per spike in bread wheat.

Association mapping was applied to identify QTLs for drought indices that were derived from GY and agro-physiological traits positively and strongly correlated (*p* < 0.001) with GY as an alternative selection approach to improve drought tolerance in wheat. Therefore, the objectives of this study were to detect MTAs and QTLs significantly associated with drought indices and to identify drought-tolerant as well as stable genotypes from a durum wheat study panel.

## Materials and Methods

### Study Panel

The study panel included 215 Ethiopian durum wheat landraces, 10 released durum wheat varieties and 10 advanced durum wheat lines from the Ethiopian Institute of Agricultural Research (EIAR), and 50 durum wheat lines from the International Wheat and Maize Improvement Centre (CIMMYT) (Negisho et al., 2021).

### Field Experiments

The field phenotyping experiments were conducted in four locations for three seasons (2016–2018) in Ethiopia. The locations were grouped into two moisture variants, field drought stress (FDS) and field non-stress (FNS). An incomplete block alpha lattice design with 3 replications per location per accession was used. A detailed summary of field experiments and evaluated traits was presented in the study of Negisho et al. (2022).

### Drought Indices Analysis

Phenotypic traits with significant (*p* < 0.05) ANOVA results for accessions, treatments, and accessions x treatment interaction, with moderate (52.25%) to high (74.91%) heritability for HI and SPS, respectively, were used. Also, traits with positive and significant (*p* < 0.001) correlation with GY under FDS and FNS conditions were selected to calculate drought indices (Negisho et al., 2022). These traits were GY, DGF, SPAD, SPS, HI, and TKW. Data across years and locations per FNS and FDS were combined to analyze the lsmeans. The lsmeans of these traits were estimated for each accession using the lme4 package in R (Lenth, 2016). Variance components of selected traits were computed by restricted maximum likelihood following the model of Yu et al. (2006). Then, drought indices (DSI, GMP, RDI, STI, TOL, and YSI) were calculated from lsmeans values of these traits. Pearson correlation coefficients (*r*) were analyzed by cor and Corstars function in R and plotted by the R package “corrplot” (Wei et al., 2017). Principal component analysis (PCA) for the drought tolerance indices was analyzed by R package FactoMineR (Husson et al., 2010). The description of drought indices and their corresponding equation are indicated in Table 1.

**Table 1:** Drought indices calculated from grain yield and from traits with a significant positive correlation (*p* < 0.001) with grain yield (GY) under FDS and FNS conditions.

**Drought indices**	**Formula (equation)**	**References**
Drought susceptibility index (DSI)	[1-(GY_FDS/GY_FNS)]/[1–(${\bar{Y}}_{\text{FDS}}/{\bar{Y}}_{\text{FNS}}$)]	Fischer and Maurer, 1978
Relative drought index (RDI)	(*GY_FDS/*GY_FNS)/(${\bar{Y}}_{\text{FDS}}/{\bar{Y}}_{\text{FNS}}$)	Fischer et al., 1998
Stress tolerance index (STI)	(GY_FDS × GY_FNS)/(${\bar{Y}}_{\text{FNS}}^{2}$)	Fernandez, 1992
Geometric mean productivity (GMP)	$\sqrt{\text{GY}\_\text{FDS }\times \text{ GY}\_\text{FNS}}$	Ramirez-Vallejo and Kelly, 1998
Tolerance (TOL)	GY_FDS-GY_FNS	Rosielle and Hamblin, 1981
Yield stability index (YSI)	GY_FDS/GY_FNS	Bouslama and Schapaugh, 1984

*GY_FDS and GY_FNS, grain yield lsmean under FDS and FNS conditions for each genotype, respectively. ${\bar{Y}}_{FDS}$ and ${\bar{Y}}_{FNS}$, grain yield lsmean under FDS and FNS conditions for all genotypes, respectively*.

### Genotyping

Genotyping was conducted by SGS Trait Genetics, Gatersleben, Germany using the wheat 90k iSelect single-nucleotide polymorphism (SNP) array (Wang et al., 2014). The consensus linkage map of tetraploid wheat (Maccaferri et al., 2015) and the IWGSC RefSeq v1.0 genomic assembly (International Wheat Genome Sequencing Consortium, 2018) were applied to assign a genomic location to each SNP marker. SNP markers with minor allele frequency (MAF) of < 5%, missing data > 10%, and heterozygosity >12.5% were omitted, and SNP markers were imputed by the Beagle software package in R (Browning and Browning, 2007). A total of 11,919 SNPs with physical positions were taken from the reference sequence of durum wheat (Maccaferri et al., 2019). Population structure and genome-wide association study (GWAS) were taken from our previous study (Negisho et al., 2021). STRUCTURE HARVESTER (Earl and vonHoldt, 2012) was used to determine the q-matrix based on the results obtained for population structure by the STRUCTURE 2.3.4 software (Evanno et al., 2005). Linkage disequilibrium (LD), LD decay, and LD plots within and across chromosomes of durum wheat genomes (A and B) were analyzed using R packages “genetics,” “LDheatmap,” and “trio” (Shin et al., 2006; Warnes, 2013; R Development Core Team, 2014). Inter-marker genetic distances were assessed using the consensus physical position of durum wheat with 11,919 SNPs (Maccaferri et al., 2019). The critical *r*^2^ value was set at *r*^2^ ≥ 0.2 (Voss-Fels et al., 2015; Oyiga et al., 2017).

### Genome-Wide Association Study

Genome-wide association study was conducted using the genome association and prediction integrated tool (GAPIT) in R (Lipka et al., 2012). FarmCPU method, which is iteratively using the fixed-effect model and the random effect model for powerful and efficient GWAS (Liu et al., 2016; de Souza et al., 2018), was used. MTAs were analyzed using calculated drought indices lsmeans as a phenotypic trait, filtered SNP markers, kinship matrix, and q-matrix (Yu et al., 2006). In this study, the Bonferroni correction test was too stringent to detect MTAs, thus, a threshold for significant MTAs was adjusted at –log_10_*p* ≥ 4.0 (Bai et al., 2016; Ma et al., 2016; Bhatta et al., 2018), and MTAs at FDR 5% were assessed (Benjamini and Hochberg, 1995). The PVE was calculated following (Teslovich et al., 2010). The detected MTAs were clustered into QTLs using the critical (*r*^2^ **≥** 0.2) LD decay value (4.78 Mb) (Negisho et al., 2022), and MTAs not in the LD were taken as an independent QTL (Kidane et al., 2017; Negro et al., 2019).

Based on the lsmeans of the combined analysis, each SNP locus in the MTAs with a positive phenotypic effect (a_i_ > 0) was identified as a favorable allele, and those with a negative phenotypic effect (a_i_ < 0) were identified as an unfavorable allele for the respective drought indices (Chong et al., 2019).

Even though it is difficult to make a comparison between the previously reported QTLs at the chromosomal position level, current and previous reports on QTLs related to drought indices in wheat were assessed and discussed (Dashti et al., 2007; Edae et al., 2014; Sukumaran et al., 2018; Qaseem et al., 2019; Arif et al., 2020; Ballesta et al., 2020).

### Candidate Gene Analysis

Significant (–log_10_*p* ≥ 4.0) MTAs for drought index traits were aligned with the annotated sequence of Durum Wheat (cv. Svevo) RefSeq Release 1.0 at GrainGenes (Maccaferri et al., 2019). In addition, detected MTAs were further assessed for their association with drought tolerance using previously published literature. Finally, in case, the annotation is not found in Durum Wheat (cv. Svevo) RefSeq Release 1.0 at GrainGenes and also not reported so far in the previously published literature, and then, the detected MTAs were considered as novel.

## Results

**Figure 1:**
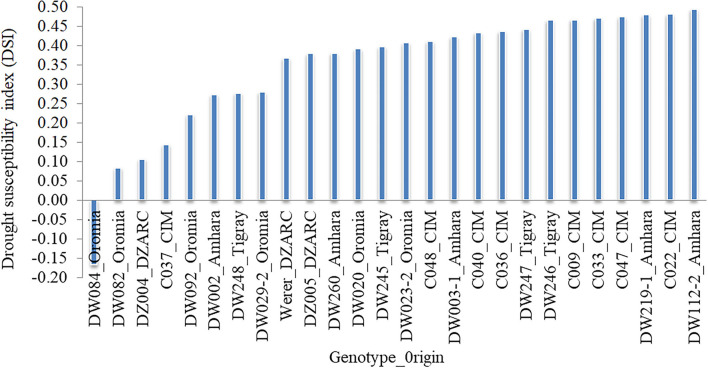
Top 26 drought tolerant accessions identified based on the drought susceptibility index calculated from grain yield (GY). The *x*-axis indicates selected genotypes and seed origin with DSI < 0.5 and the *y*-axis shows DSI values.

Mean grain yield under field non-stress (GY_FNS) and field drought stress (GY_FDS) conditions were 77.09 and 49.5 g/plot showing 35.79% GY reduction with 20.25 and 23.25% coefficient of variation, respectively (Table 2). The mean values of drought indices were 0.97, 1.01, 0.66, 61.49, 27.61, and 0.65 for DSI, RDI, STI, GMP, TOL, and YSI, respectively. Deviation of the data from the mean was expressed in percentage of standard deviation (SD%). A higher percentage of SD was observed for GY under FNS (15.61%) as compared to FDS (11.49%). Similarly, a higher percentage value of SD was detected for GMP (12.13%) and TOL (12.33%) as compared to the other drought indices. The coefficient of variation for the drought indices ranged from 19.27 (GY-STI) to 44.64% (GY-TOL) (Table 2). The 52% of the accessions (147) in the SP revealed GY-DSI values <1 that indicates the existence of drought-tolerant accessions. Out of drought-tolerant accessions, 96 were from Ethiopian durum wheat landraces, 9 from advanced lines, 7 from released varieties, and 35 were from the CIMMYT durum wheat collection, and the top 26 (9%) are visualized in Figure 1. DW084, DW082, DZ004, C037, and DW092 were selected as the top five drought-tolerant accessions based on the combined rank of GY-DSI, GY-RDI, GY-TOL, and GY-YSI (Figure 1, Supplementary Table S1). Additionally, accessions with high value based on the combined rank of GY-GMP and GY-STI are considered as stable genotypes under FDS and FNS. Based on GMP and STI drought indices ranking C010, DW033, DW080, DW124-2, and C011 were selected as the top five stable accessions. The remaining 138 (48%) accessions in the SP showed a GY-DSI value higher than one indicating the susceptibility of the accessions to drought.

**Table 2:** Descriptive statistics for grain yield (GY) drought indices.

**Traits**	**Mean**	**SD%**	**Min**	**Max**	**%CV**
GY_FNS	77.09	15.61	32.75	114.63	20.25
GY_FDS	49.50	11.49	23.50	79.92	23.21
% GY loss	35.79	–	–	–	–
DSI	0.97	0.35	–0.17	1.86	36.05
RDI	1.01	0.20	0.52	1.65	19.33
STI	0.66	0.25	0.17	1.37	37.27
GMP	61.49	12.13	32.05	90.27	19.72
TOL	27.61	12.33	1.24	61.11	44.64
YSI	0.65	0.13	0.34	1.06	19.31

*GY_FNS, grain yield lsmeans in g/plot under FNS; GY_FDS, grain yield lsmeans in g/plot under FDS; %GY loss, percentage of yield loss due to drought stress; DSI, drought susceptibility index; RDI, relative drought index; STI, stress tolerance index; GMP, geometric mean productivity; TOL, tolerance index; YSI, yield stability index*.

*Mean, standard deviation (SD), minimum, maximum, and percentage of the coefficient of variation (CV), n = 285*.

### Correlation Analysis

**Figure 2:**
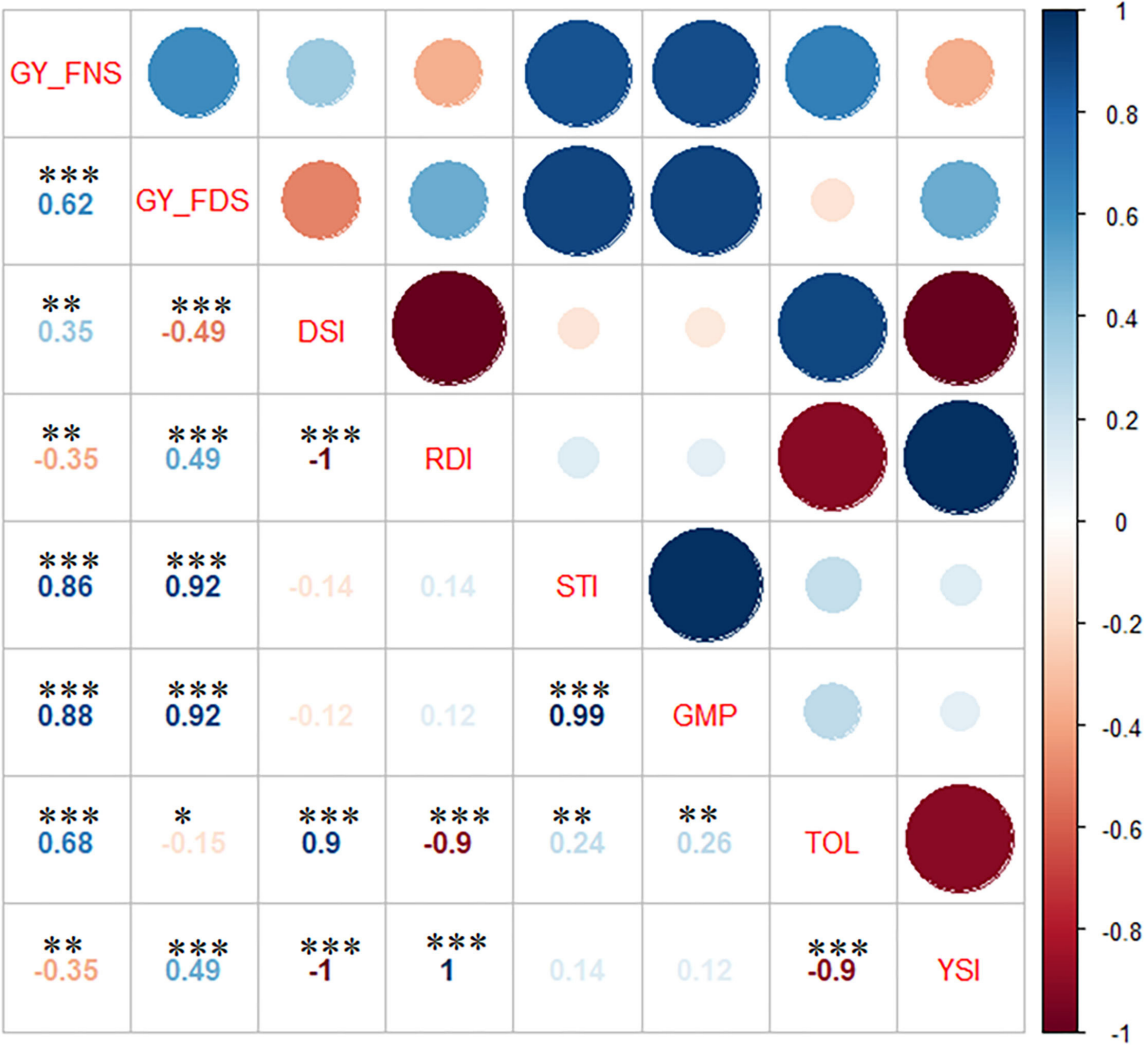
Pearson correlation between the drought indices traits. GY_FNS, lsmeans from FNS at Holeta and Debre Zeit; GY_FDS, lsmeans from FDS at Dera and Melkassa; DSI, Drought susceptibility index; RDI, relative drought index; STI, stress tolerance index; GMP, geometric mean productivity; TOL, tolerance index; YSI, yield stability index. ^*^, ^**^, and ^***^ significance at *p* < 0.05, *p* < 0.01, and *p* < 0.001, respectively.

A significant positive correlation was observed between GY_FNS and GY_FDS (*r* = 0.62) (Figure 2). Likewise, GY_FNS and GY_FDS were significantly and positively correlated with GMP (*r* = 0.88 and 0.92) and STI (*r* = 0.86 and 0.92), respectively.

GY_FNS was significantly and positively correlated with DSI (*r* = 0.35) and TOL (*r* = 0.68), but a significant (*r* = −0.35) negative correlation was observed with RDI and YSI. GY_FDS was significantly (*r* = 0.49) and positively correlated with RDI and YSI but significantly and negatively correlated with DSI (*r* = −0.49) and TOL (*r* = −0.15). There was a significant positive correlation between DSI and TOL (*r* = 0.9). A highly significant (*r* = −1.0) negative correlation was observed between RDI and YSI. RDI was significantly and positively correlated with YSI (*r* = 1.0) but showed a strong significant negative correlation with TOL (*r* = −0.9). STI and GMP showed a significant (*r* = 0.99) positive correlation. STI and GMP revealed a significant positive correlation with TOL (0.24 and 0.26), respectively. Finally, there was a strong significant negative (*r* = −0.9) correlation between TOL and YSI.

### PCA

**Figure 3:**
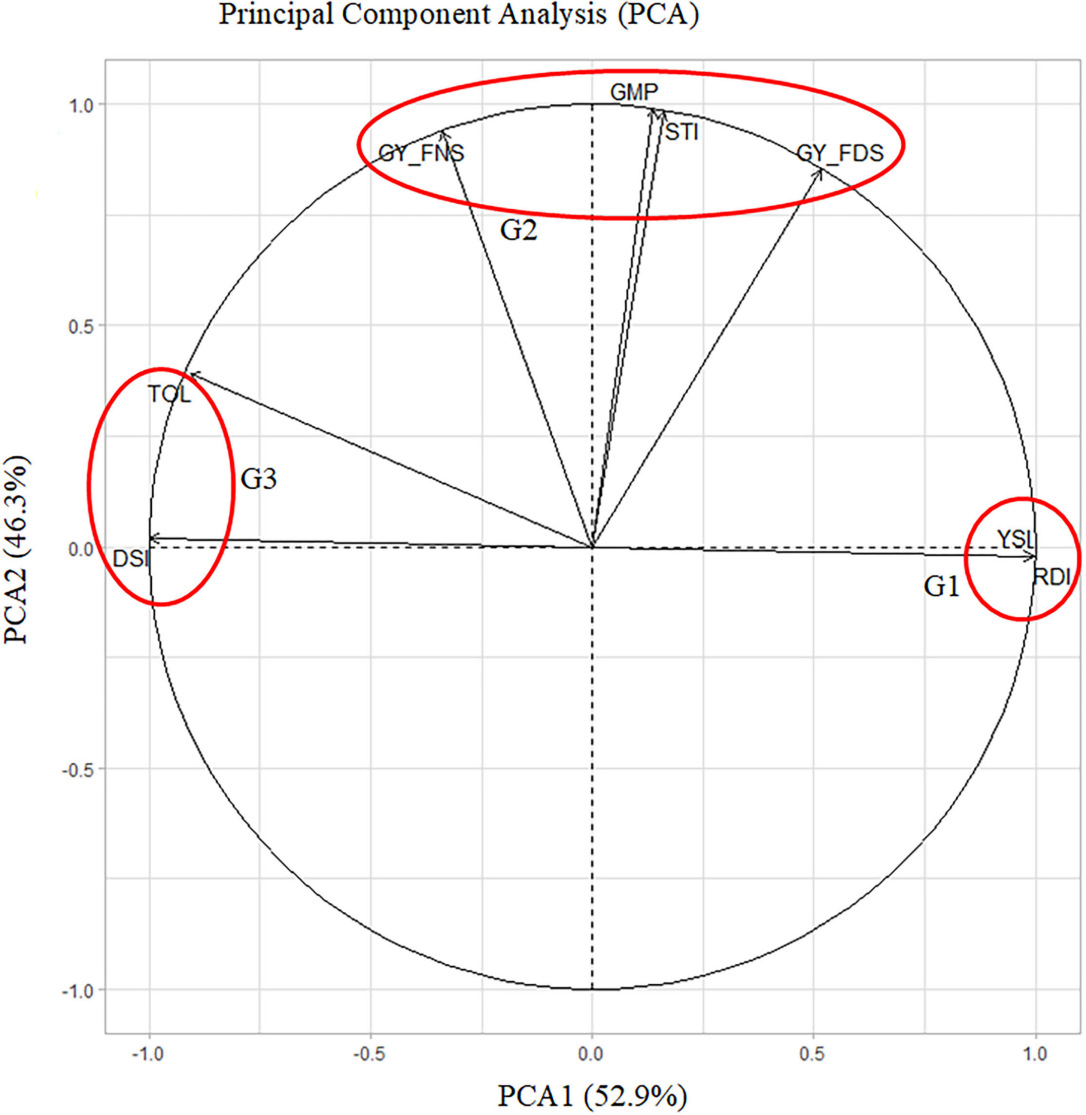
PCA showing the contribution of drought indices. PCA1 and PCA2 accounted for 99.2% of total variations among drought indices.

PCA1 and PCA2 explained 52.9 and 46.3% of the variation among drought indices, respectively (Figure 3). PCA clustered the drought indices into three groups (G1, G2, and G3). G1 indicated drought-tolerant accessions with higher values of YSI and RDI, G2 indicated stable accessions with higher values of GY_FNS, GY_FDS, GMP, and STI, and G3 showed drought-tolerant accessions with lower values of DSI and TOL. A narrow angle (<90°) shows a positive correlation within each group, whereas a wide angle (>90°) indicates a negative correlation, e.g., between G1 and G3. Hence, GY_FNS was positively correlated with GY_FDS, STI, GMP, TOL, and DSI, but negatively correlated with YSI and RDI. Similarly, GY_FDS was positively correlated with GY_FNS, YSI, RDI, STI, and GMP but negatively correlated with DSI and TOL as was revealed by Pearson correlation analysis (Figure 2; Supplementary Figure S1).

### Marker-Trait Association Analysis for Drought Indices

A total of 184 MTAs were detected across the durum wheat genome for the analyzed drought indices at –log_10_*p* ≥ 4.0 (Table 3) explaining up to 26.61% of the total phenotypic variation. The Manhattan plots for MTAs were indicated in Supplementary Figures S2–S7. A total of 41 (22.28%) of the significant MTAs detected were associated with two or more drought indices highlighted in blue color (Supplementary Table S2). Predominantly, 16 (39.02%) of these stable MTAs were associated with GMP and STI drought indices.

**Table 3:** Significant (–log_10_*p* ≥ 4.0) marker-trait associations (MTAs) and quantitative trait loci (QTL) that were detected for the drought indices traits calculated from grain yield and traits significantly (*p* < 0.001) positively correlated with grain yield under FNS and FDS.

**Trait**	**MTA**	**MTAs per chromosomes**	**QTL**
GY-DSI	0	—	0
GY-GMP	10	2A, 2B, 3B, 4A, 5A, 5B, 6B, 7A (2), 7B	6
GY-RDI	6	1A (2), 1B, 4A, 7A, 7B	4
GY-STI	8	1A, 2A, 3B, 4B, 5A (2), 5B, 6B	4
GY-TOL	0	—	0
GY-YSI	0	—	0
DGF-DSI	3	1A, 3B, 4B	1
DGF-GMP	2	1A, 4B	0
DGF-RDI	7	1A, 1B, 3B,4B, 5B, 6B (2)	5
DGF-STI	5	1B, 2A, 2B, 3B, 7B	4
DGF-TOL	6	3B, 4A, 4B, 5B, 6B, 7A	4
DGF-YSI	6	2B, 3B, 4B, 5B, 6B (2)	2
SPAD-DSI	2	1A, 2B	1
SPAD-GMP	11	1A (2), 3A, 3B, 4A, 4B (2), 5A, 6B (2), 7A	8
SPAD-RDI	2	1A (2), 1B, 2B (2), 4B, 5A, 6B (2)	2
SPAD-STI	7	1A, 1B, 2B (2), 4B, 6B (2)	6
SPAD-TOL	0	—	0
SPAD-YSI	4	1A (2), 2B, 3A	2
SPS-DSI	2	1B, 2B	1
SPS-GMP	10	1A, 2A (3), 2B, 3A, 5A, 5B, 6A, 7A	6
SPS-RDI	2	1B, 2B	0
SPS-STI	9	1A (2), 2A, 3A, 3B, 4B, 5A 7A (2)	8
SPS-TOL	0	—	0
SPS-YSI	2	1B, 2B	0
HI-DSI	8	1A, 1B, 2A (2), 4B, 6A, 6B, 7A	6
HI-GMP	11	1A (2), 1B, 2B, 4A, 4B, 5A (2), 7A (2), 7B	3
HI-RDI	6	2A (2), 4B, 6B, 7A, 7B	1
HI-STI	7	1B, 4A, 4B, 5A, 7A, 7B (2)	5
HI-TOL	4	2A (2), 4B, 6B	2
HI-YSI	6	2A (2), 4B, 6B, 7A, 7B	3
TKW-DSI	8	2B (2), 4A (2), 4B (2), 6B, 7A,	4
TKW-GMP	6	1A, 2B (2), 4A, 4B, 6A	4
TKW-RDI	8	2B (2), 4A (2), 4B, 7A, 7B (2)	1
TKW-STI	4	2B, 4A, 6A, 7A	1
TKW-TOL	5	2A, 2B, 5A, 5B, 7B	5
TKW-YSI	7	2B, 4A (2), 4B, 6B, 7A (2)	3
Total	184	—	102
Genome		Detected MTAs: A = 89 (48%) and B = 95 (52%) Detected QTLs: A = 48 (47%) and B = 54 (53%)	

*Brackets enclose the number of MTAs detected per chromosome only if it is more than one*.

In this study, SNP alleles with positive effects that led to increased drought index traits were defined as “favorable alleles.” Accordingly, five major MTAs were detected associated with GY-GMP as favorable SNP alleles (>10% PVE): RFL_Contig2569_2187 on chromosome 3B at 752,249,328 bp, Kukri_c22602_704 on chromosome 4A at 733,371,835, IAAV2346 on chromosome 5B at 17,863,862 bp, wsnp_Ex_c3940_7144946 on chromosome 6B at 508,076,861 bp, and Tdurum_contig4658_346 on chromosome 7B at 663,797,774 bp. On the other hand, four major MTAs were detected associated with GY-GMP as unfavorable SNP alleles: Tdurum_contig10785_2433 on chromosome 2A at 12,102,513 bp, Kukri_rep_c116526_98 on chromosome 5A at 112,213,041 bp, BobWhite_C21378_234 on chromosome 7A at 693,389,984 bp, and wsnp_Ex_c5839_10246915 on chromosome 7A at 709,145,347 bp. From these, three major MTAs with favorable SNP alleles located on chromosomes 2B, 5B, and 7B, and two major MTAs with unfavorable SNP alleles located on chromosomes 7A were novel MTAs. Generally, in this study, the phenotypic effect size on drought indices ranged from −5 to 5 (Supplementary Table S2).

#### Candidate Genes

Candidate genes for MTAs linked with drought tolerance were calculated from grain yield with identified positive phenotypic effect size, particularly U-box domain-containing protein on chromosome 4A associated with GY-GMP, potassium transporter on chromosome 3B associated with GY-GMP, MODIFIER OF SNC1 1 G on chromosome 5A associated with GY-GMP, and cytochrome P450 family protein on chromosome 7A associated with GY-RDI. As regards the MTAs associated with drought indices calculated from DGF, the genes identified were methyltransferase on chromosome 4A and leucine-rich repeat receptor-like kinase (LRK2) on chromosome 6B associated with DGF-TOL. In this study, other important MTAs identified associated with drought tolerance were as follows: UNC93-like protein on chromosome 5A associated with SPAD-GMP, ribosomal protein on chromosome 4B associated with HI-TOL, HI-RDI, HI-YSI, Acyl-CoA dehydrogenase-related family protein on chromosome 2B associated with TKW-TOL, and RNA-binding protein on chromosome 1A associated with HI-DSI.

### MTA Cluster Into QTL

**Figure 4:**
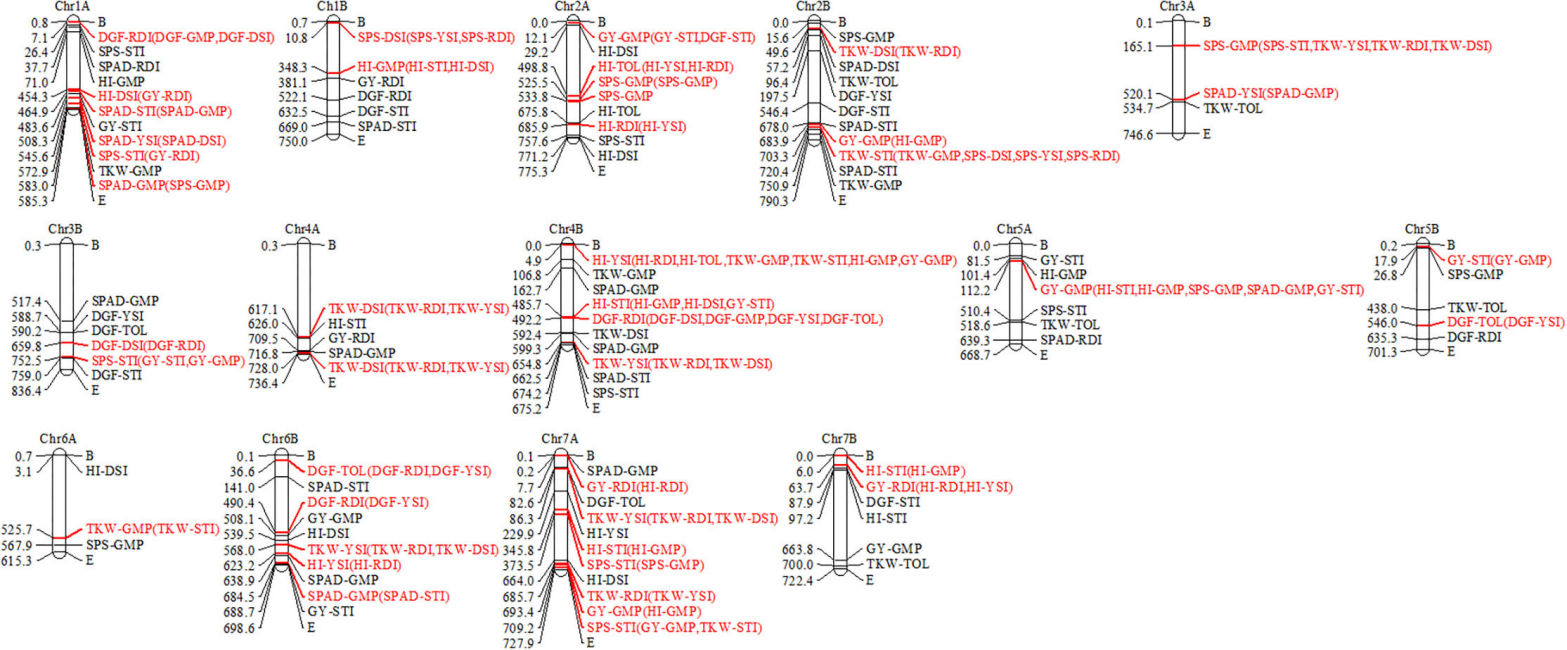
Linkage map showing number of QTLs detected for drought indices. Co-clustered QTLs were marked in red color and in parenthesis, and individual QTLs were marked in black color.

The detected MTAs for drought tolerance indices were clustered into 102 QTLs (Supplementary Table S3). The numbers of QTLs detected from the highest to the lowest were 28, 27, 13, 13, 11, and 10 for STI, GMP, DSI, RDI, TOL, and YSI drought indices, respectively (Supplementary Table S3; Figure 4). Out of which, 43 stable QTLs harbor more than one drought tolerance index (up to four drought indices), for instance, four drought indices QTLs were co-located on chromosome 4B between 487,222,406–497,250,660 and 4,927,519–9,941,646 bp shown in red color. In contrast, some detected QTLs like those located on chromosome 1A between 478,563,347–488,591,601 and 66,026,146–76,054,400 bp are examples of individual QTLs for STI and GMP, respectively, as indicated by black color (Figure 4).

A total of twenty-eight detected QTLs for STI were calculated from SPS, SPAD, GY, DGF, TKW, and HI traits, out of which, ten stable QTLs are co-located with QTL for GMP, DSI, RDI, and YSI located on chromosomes 1A, 2B, 3B, 4B, 5A, 7A, and 7B. The remaining 18 were individual QTLs for STI. Out of 28 selected QTLs for STI located on chromosomes 1A (3 QTLs), 2B (3 QTLs), 3B (2 QTLs), 4B (3 QTLs), 5A, 6B, and 7B (3 QTLs), 16 were not reported so far and are likely to be novel. A total of twenty-seven QTLs were detected for GMP calculated from HI, TKW, SPAD, GY, SPS, and DGF traits, out of which, ten stable QTLs were co-located with QTL for STI, DSI, YSI, and RDI on chromosomes 1A, 1B, 2A, 2B, 3A, 5A,6A, 6B, and 7A, whereas the other 17 detected QTLs were individual QTLs for GMP. Out of the 27 detected QTLs for GMP, 26 could be novel.

As regarded the 13 detected QTLs for DSI were calculated from HI, SPS, TKW, SPAD, DGF, and GY traits, of which six stable QTLs are co-located with QTL for RDI and YSI on chromosomes 1A, 1B, 2B, 3B, and 4A. The remaining seven were individual QTLs for DSI. From the six co-located QTLs for DSI, three QTLs were associated with RDI located on chromosomes 1A, 2B, and 3B between 449,301,491–459,329,745, 44,634,623–54,662,877, and 654,733,402–664,761,656 bp, respectively. Similarly, DSI QTLs were co-located with RDI and YSI located on chromosomes 1B between 5,764,433–15,792,687 bp and on 4A between 722,943,476–732,971,730 and 612,075,413–622,103,667 bp. Seven QTLs detected for DSI located on chromosomes 1A, 1B, 2A (2 QTLs), 2B, 3B, and 6A were not reported so far and are novel putatively QTLs.

The 13 detected QTLs for RDI were calculated from DGF, SPAD, GY, HI, and TKW traits, of which seven stable QTLs included were co-located with GMP, DSI, YSI, and STI on chromosomes 1A, 2A, 4B, 6B, 7A, and 7B, whereas the others six detected were individual QTLs. The ten QTLs detected for RDI, located on chromosomes 1A (2 QTLs), 1B (2 QTLs), 2A, 5A, 6B, 7A (2 QTLs), and 7B, were could be new.

The 11 detected QTLs for TOL were calculated from HI, TKW, and DGF. Three QTLs are co-located with QTL for YSI and RDI on chromosomes 2A, 5B, and 6B, and the remaining eight are individual QTLs for TOL. In particular, five QTLs for TOL that were located on chromosomes 2A (2 QTLs), 4A, 6B, and 7A, were putatively novel.

The 10 detected QTLs for YSI were calculated from SPAD, DGF, HI, and GY, of which seven QTLs located on chromosomes 1A, 3A, 4B, 6B, and 7A were co-located with QTLs for DSI, GMP, RDI, TOL, and STI. The remaining eight QTLs for YSI were likely to be new.

**Figure 5:**
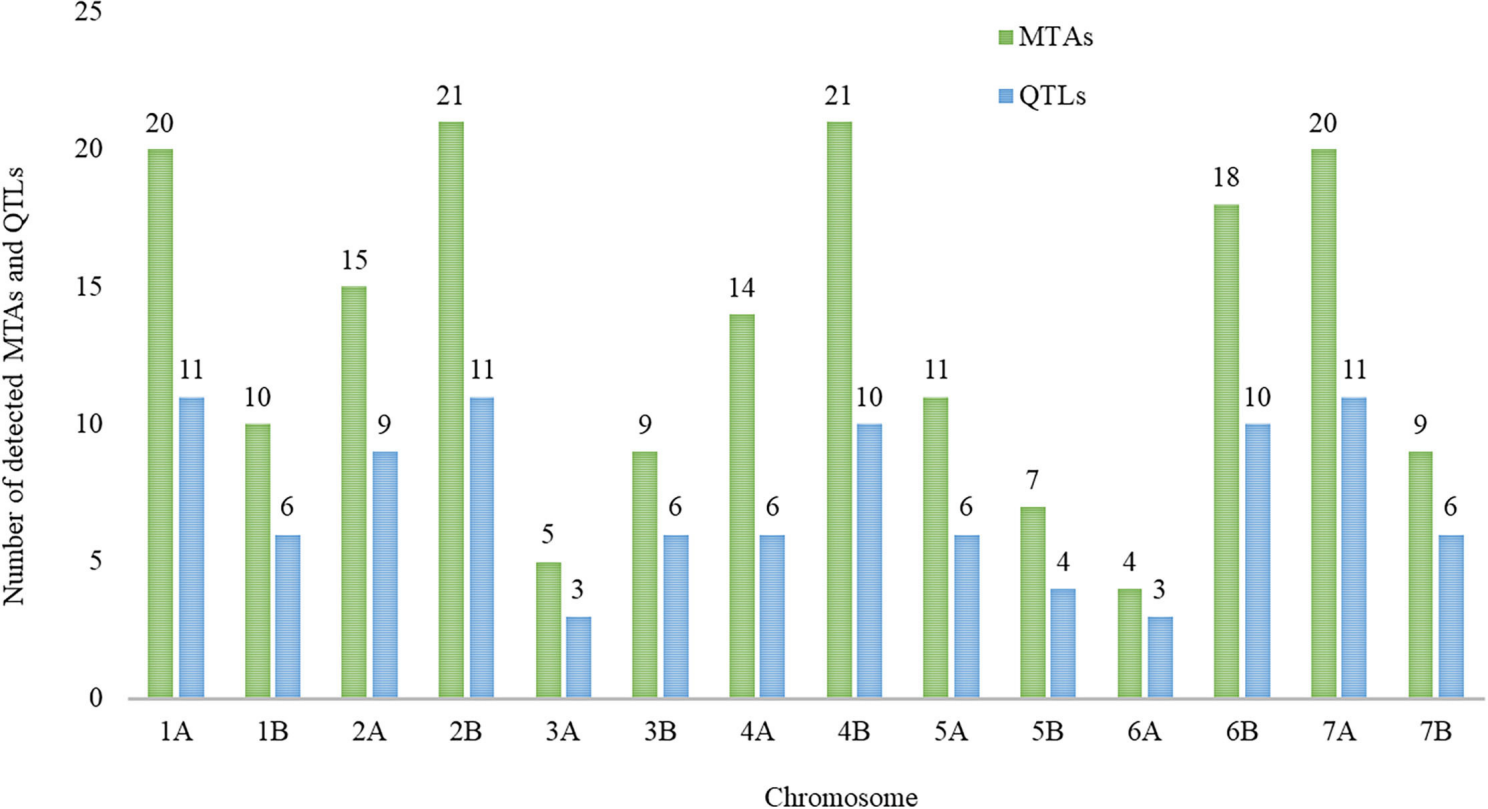
The number of detected marker-trait associations (MTAs) and quantitative trait loci (QTLs) that were detected for drought indices across the durum wheat genome.

The distribution of single MTA/QTL on genomes A and B was 48%/47% and 52%/53%, respectively (Table 3). Chromosomes 1A, 2B, and 7A each harbor eleven QTLs, which is the highest number of QTLs detected per chromosome followed by ten QTLs each were detected on chromosomes 4B and 6B, and nine QTLs were detected on chromosome 2A (Figure 5). In our study, these chromosomal regions were considered as QTL hotspots for drought tolerance in durum wheat. The lowest numbers of QTLs (3) each were detected on chromosomes 3A and 6A. Six (5.88%) of the detected QTLs were major QTLs and all of them were associated with GY-GMP drought index between 658,783,647–668,811,901, 503,062,734–513,090,988, 107,198,914–117,227,168, 678,867,539–688,895,793, 7,088,386–17,116,640, and 688,375,857–698,404,111 bp and located on chromosomes 7B, 6B, 5A, 2B, 2A, and 7A with 22.21, 21.91, 17.00, 14.72, 14.59, and 13.59% PVE, respectively (Supplementary Table S3).

## Discussion

Drought tolerance is a complex quantitative trait, which is affected by the timing and severity of drought stress relative to plant development and growth. In this study, 35.79% GY reduction was observed under field conditions in durum wheat due to drought stress. In agreement with this, depending on plant growth stage and severity of drought, 60% in durum wheat (Sukumaran et al., 2018) and 10–76% grain yield reduction in bread wheat have been reported (Grzesiak et al., 2019). Nevertheless, studies revealed that in wheat, the effect of drought stress is more pronounced during the reproductive stage (Nezhadahmadi et al., 2013). In our study, 147 (52%) accessions from the study panel revealed a GY-DSI value of <1, indicating drought tolerance, whereas 138 (48%) showed a GY-DSI higher than 1, implying that these genotypes are drought susceptible. This suggests that in this study, drought stress was moderate but enough to facilitate the selection of drought-tolerant accessions. Moderate drought stress was reported as recommended to select drought-tolerant wheat lines (Ali and El-Sadek, 2016; Patel et al., 2019).

From our previous experimental procedures under FDS and FNS conditions, traits from which drought indices were calculated showed significant (*p* < 0.05) differences among durum wheat accessions, between treatments, and for accession x treatment interaction. This illustrates the broad genetic diversity present in the panel herein used for drought tolerance in general and in Ethiopian durum wheat landraces in particular (Negisho et al., 2021). Also, moderate to high heritability values and a significant (*p* < 0.001) correlation with GY under FDS and FNS conditions have been found in this study. This relation provides the basis for utilizing drought tolerance indices as a means to explain the phenotypic variation. It has been also reported that drought tolerance indices can be derived from GY and traits that are strongly and positively correlated with GY as a measure for selecting the best genotypes (Farshadfar et al., 2012; Patel et al., 2019; Ayed et al., 2021).

The significant (*r* = 0.62) positive correlation between GY_FNS and GY_FDS suggests that high GY performance under the FNS condition is generally closely connected with stable and high GY under FDS conditions. Similarly, studies depicted a positive and significant correlation between GY under favorable and drought stress conditions in durum wheat (Patel et al., 2019), bread wheat (Ali and El-Sadek, 2016), and rice (Mau et al., 2019). The strong and positive correlation of GY_FNS and GY_FDS: GMP (*r* = 0.88 and 0.92) and STI (*r* = 0.86 and 0.92), respectively, suggests that GMP and STI may be potential drought indices to select stable and relatively higher-yielding accessions under drought stress conditions. Respectively, GMP and STI were reported as convenient drought indices parameter to select stable and high-yielding durum wheat genotypes under drought stress and non-stress conditions (Patel et al., 2019; Ayed et al., 2021). Interestingly, in this study, three out of the top five accessions selected based on the combined rank of drought indices were from Ethiopian durum wheat landraces and could be recommended as parents for wheat drought-tolerant improvement breeding with other cultivars.

The first two PCAs explained 99.2% of the total variation among drought indices and clustered the drought indices into three groups, G1 indicating drought-tolerant accessions with high values of RDI and YSI, G2 indicating yield-stable and drought-tolerant accessions with high values of GY_FNS, GY_FDS, GMP, and STI, and G3 indicating drought-tolerant accessions with lower values for DSI and TOL. The PCA angles in our study also allowed us to interpret the interrelationships among the drought indices and were confirmed with correlation analysis and scatter plot results (Figure 3; Supplementary Figure S1).

In this study, a total of 102 QTLs were detected at –log_10_*p* ≥ 4.0. The number of QTLs on A and B genomes was (48%) and (52%), respectively. In accordance, research results showed a larger number of QTLs on the B genome as compared to the A genome in durum wheat (Soriano et al., 2017; Desiderio et al., 2019; Alemu et al., 2020; Ballesta et al., 2020). Similarly, using simple sequence repeat (SSR) and diversity array technology (DArT) markers, Maccaferri et al. (2014) mapped a higher number of markers on the B genome as compared to the A genome. Our result showed at the chromosomal level, a higher number of QTLs (11.78%) each were located on chromosomes 1A, 2B, and 7A, suggesting that these genome regions are QTL hotspots and play a pivotal role in drought tolerance in wheat. In this study, a considerable number of QTLs, namely, 6 (5.88%), were detected for drought indices on chromosome 4A, which is in agreement with the result reported by Ballesta et al. (2020).

In our study, six of the 13 QTLs detected for DSI were on chromosomes 2B, 4A (2 QTLs), 4B, 6B, and 7A between 44,634,623–54,662,877, 612,075,413–622,103,667, 722,943,476–732,971,730, 587,392,128–597,420,382, 534,453,653–544,481,907, and 658,941,965–668,970,219 bp, respectively (Supplementary Table S3). Accordingly, studies reported QTLs for DSI located on chromosomes 2B, 4A (2 QTLs), 4B, 6B, and 7A (Dashti et al., 2007; Edae et al., 2014; Sukumaran et al., 2018; Ballesta et al., 2020). To the best of our knowledge, seven QTLs detected for DSI located on chromosomes 1A, 1B, 2A (2 QTLs), 2B, 3B, and 6A between 449,301,491–459,329,745, 5,764,433–15,792,687, 24,208,804–34,237,058, 766,212,336–776,240,590, 52,165,550–62,193,804, 654,733,402–664,761,656, and 3,084,526–80,98,653 bp were not reported so far and could be novel.

Edae et al. (2014) detected QTLs for SPS-DSI located on chromosomes 7A and 7B using DArT markers. However, we did not find QTL for SPS-DSI on these chromosomes. In this study, QTLs for SPS-STI were detected on chromosomes 1A, 2A, 3B, 4B, 5A, and 7A. Edae et al. (2014) also detected QTL GY-DSI located on chromosome 4A, but no QTL was detected for GY-DSI on this chromosome.

In this study, three out of the 13 QTLs for RDI were detected on chromosomes 4A, 4B, and 5B between 704,477,416–714,505,670, 487,222,406–497,250,660, and 630,323,029–64,0351,283 bp, respectively. Similarly, studies reported QTLs for RDI on chromosomes 4A (Arif et al., 2020) and 4B (Ballesta et al., 2020) and chromosome 5B (Arif et al., 2020). The other QTLs detected for RDI could be new. A QTL was detected associated with GMP on chromosome 3B between 512,345,933–522,374,187 bp. In agreement with, Dashti et al. (2007) reported a QTL on chromosome 3B using SSR marker in doubled haploid bread wheat associated with GMP. In our study, 12 of the 28 detected QTLs for STI were on chromosomes 1A, 2B (2 QTLs), 2A, 2B, 4A, 5A, 5B, 6B, and 7A (2 QTLs) between 478,563,347–488,591,601, 627,518,169–637,546,423, 663,977,245–674,005,499, 752607375–762,635,629, 698,319,457–708,347,711, 621,025,922–631,054,176, 76,490,700–86,518,954, 12,849,735–22,877,989, 683,730,386–693,758,640, 340,762,156–350,790,410, 368,439,457–378,467,711, and 704,181,285–714,209,539 bp, in that order. In agreement with this, studies in wheat QTLs were reported for STI on chromosomes 1A, 1B, 2A, 2B, 4A, 5A, 5B, 6B, and 7A (Dashti et al., 2007; Sukumaran et al., 2018; Qaseem et al., 2019; Arif et al., 2020; Ballesta et al., 2020). The remaining 18 detected QTLs for STI were likely to be novel QTLs.

Moreover, six out of the 11 QTLs identified for TOL were detected on chromosomes 2B, 3A, 5A, 5B (2 QTLs), and 7B between 91,393,993–101,422,247, 529,677,366–539,705,620, 513,570,349–523,598,603, 433,014,029–443,042,283, 540,970,848–550,999,102, and 695,007,223–705,035,477 bp, respectively. Consistent with this result, studies revealed QTLs for TOL located on chromosomes 2B, 3A, 5A, 5B, and 7B (Dashti et al., 2007; Sukumaran et al., 2018; Arif et al., 2020; Ballesta et al., 2020). However, five of the 11 detected QTLs for TOL located on chromosomes 2A (2 QTLs), 4A, 6B, and 7A were not reported so far and could be novel. Out of the detected 10 QTLs for YSI, two were located on chromosomes 4B and 6B between 649,804,818–659,833,072 and 563,024,848–573,053,102 bp, correspondingly. Similarly, Ballesta et al. (2020) reported QTLs for YSI on chromosomes 4B and 6B, whereas the remaining eight are likely new QTLs.

In general, 30 out of the 102 detected QTLs for drought indices were previously reported (Dashti et al., 2007; Sukumaran et al., 2018; Qaseem et al., 2019; Arif et al., 2020; Ballesta et al., 2020), whereas 72 QTLs reported in this study are likely novel QTLs.

In this study, MTAs that were previously reported associated with drought stress tolerance and/or their annotation show associations with drought stress tolerance are considered as candidate genes. Accordingly, one MTA was identified associated with GY-GMP on chromosome 4A (Kukri_c22602_704) at 733,371,835 bp, annotated as a U-box domain-containing protein. In agreement, studies indicated the involvement of these proteins in drought stress in barley (Ryu et al., 2019) and in drought and salinity stresses in Arabidopsis (Cho et al., 2006). Another MTA was detected associated with GY-GMP on chromosome 6B (wsnp_Ex_c3940_7144946) at 508,076,861 bp, annotated as a DNA topoisomerase 2. Similarly, studies showed the upregulation of DNA topoisomerase 2 under abiotic stresses, such as cold and salinity in tobacco and pea (John et al., 2016; Tammaro et al., 2016). An MTA was detected associated with GY-GMP on chromosome 3B (RFL_Contig2569_2187) at 752,249,328 bp, annotated as a potassium transporter. Congruent to this, Ouyang et al. (2010) and Cheng et al. (2018) reported overexpression of a potassium transporter (OsHAK1) in rice enhanced drought tolerance at both vegetative and reproductive stages *via* decreasing the levels of lipid peroxidation, increasing proline accumulation, and improving the activities of antioxidant enzymes. One MTA was detected on chromosome 5A (Kukri_rep_c116526_98) associated with GY-GMP at 112,213,041 bp, annotated as a Protein MODIFIER OF SNC1 1 G. In line with this, the research report showed the involvement of MOS14 (protein modifier of snc1-1, 14) in drought tolerance in Arabidopsis (Xu et al., 2016). As regards, the MTA was detected associated with GY-GMP and GY-STI on chromosome 2A (Tdurum_contig10785_2433) at 12,102,513 bp, annotated as an NBS-LRR-like resistance protein. It is known that NBS-LRR-like resistance protein is particularly involved in resistance to various diseases (Shao et al., 2014; Dubey and Singh, 2018), as well as in drought stress tolerance (Chini et al., 2004; Rampino et al., 2017).

An MTA was identified associated with GY-RDI and HI-DSI on chromosome 1A (Ra_c2895_591) at 454,315,618 bp, annotated as an RNA-binding protein (RBP). Similarly, Marondedze et al. (2019) reported that RBPs operate as a post-transcriptional modulator in drought stress in Arabidopsis by controlling the stability of metabolic processes for short and long-term stress adaptations. The other MTA was detected associated with GY-STI, HI-GMP, HI-STI, SPAD-GMP, and SPS-GMP on chromosome 5A (Tdurum_contig76578_537) at 110,830,599 bp, annotated as UNC93-like protein. Likewise, a study indicated that UNC93 functions as a positive regulator of drought stress tolerance *via* ABA-dependent signal transduction pathways (Xiang et al., 2018). An MTA was detected associated with GY-RDI on chromosome 7A (Excalibur_c24593_1217) at 7,721,495 bp, annotated as cytochrome P450 family protein. In agreement, research reports elucidated that cytochrome P450 family protein involves in drought and salinity stresses (Narusaka et al., 2004; Ehlting et al., 2008; Jun et al., 2015). Particularly, Melloul et al. (2014) showed the upregulation of cytochrome P450 proteins in durum wheat leaves under drought stress. Another MTA was identified associated with DGF-TOL on chromosome 4A (IAAV1775) at 590,188,609 bp, annotated as a methyltransferase. Respectively, Lu et al. (2020) indicated that this protein enhances drought resistance in poplar plants by leading to a higher density of trichomes and a better-developed root system.

Marker-trait association was detected associated with DGF-RDI, DGF-TOL, and DGF-YSI on chromosome 6B (Tdurum_contig61383_627) at 36,557,072 bp, annotated as a leucine-rich repeat receptor-like protein kinase family protein. Similarly, a study on rice revealed that this family protein increases drought tolerance *via* promoting root growth while reducing plant height (Kang et al., 2017). Another MTA was detected associated with HI-RDI, HI-TOL, and HI-YSI, on chromosome 4B (tplb0050b23_546) at 4,927,519 bp, annotated as a Ribosomal protein. In agreement, research results indicated the upregulation of ribosomal proteins under drought stress in the root of drought tolerant bread wheat cultivar (Arg) (Ma et al., 2016). MTA was identified associated with TKW-TOL on chromosome 2B (Kukri_c36879_83) at 96,408,120 bp, annotated as acyl-CoA dehydrogenase-related family protein. Similarly, a study revealed that this protein is one of the drought-responsive protein species in leaves and is altered under dehydration (Wang et al., 2016).

Chromosomes 1A, 2B, and 7A are identified as QTL hotspots each encompassing 11 QTLs between 2,116,602–577,966,934, 10,629,564–745,910,537, and 172,269–704,181,285 bp, respectively (Supplementary Table S3). Despite the identification of several QTLs that were associated with drought indices in our study, further validations and investigations are needed to understand the molecular functions of the associated genes in drought stress-response mechanisms in wheat. Major QTLs with favorable SNP alleles identified in this study could be used to develop polymerase chain reaction (PCR)-based markers, such as cleaved amplified polymorphic sequence (CAPS) and competitive allele-specific polymerase chain reaction (KASP) markers, to facilitate future marker-assisted breeding in wheat.

## Conclusion

Durum wheat *Triticum turgidum* ssp. durum accessions used in our study showed large natural variation (*p* < 0.0001) for drought tolerance as assessed based on six agro-physiological traits, including GY. Among the investigated drought indices, significant correlations were observed and criteria defining drought-tolerant accessions could be defined. Based on the combined rank of GY-DSI, GY-RDI, GY-TOL, and GY-YSI, DW084, DW082, DZ004, C037, and DW092 were identified as the most drought-tolerant accessions. Similarly, based on the combined rank of GY-GMP and GY-STI, C010, DW033, DW080, DW124-2, and C011 were selected as the best stable accessions both under FDS and FNS conditions. Major MTAs with favorable SNP alleles identified in this study may be used to develop DNA markers, such as CAPS and KASP markers, for marker-assisted breeding for drought stress tolerance in wheat. The detected MTAs were further clustered into 102 QTLs. Chromosomes 1A, 2B, and 7A are QTL hotspots with 11 QTLs each. A higher number of QTLs (52%) linked to drought indices were detected on the B genome. Six (5.88%) of the identified QTLs represent major QTLs with higher than 10% PVE. The detected major QTLs were particularly associated with GY-GMP and located on chromosomes 4A, 7B, 6B, 5B, and 2B, with 22.21, 21.91, and 14.72% PVE, respectively. Our study successfully elucidated the significance and alternative means of identifying genetic loci for drought tolerance *via* drought indices using the GWAS technique.

## Data Availability Statement

The original contributions presented in the study are included in the article/Supplementary Materials, further inquiries can be directed to the corresponding author/s.

## Author Contributions

Material preparation, data collection, and analysis were performed by KN, SS, and GW. The first draft of the manuscript was written by KN and revised by AM. All authors commented on previous versions of the manuscript. All authors contributed to the study conception and design. All authors read and approved the final manuscript.

## Funding

The manuscript was part of a PhD thesis (KN) financially supported by the Federal Ministry of Food and Agriculture [Bundesministerium für Ernährung und Landwirtschaft (BMEL), Germany (FKZ 2813FS01)].

## Conflict of Interest

The authors declare that the research was conducted in the absence of any commercial or financial relationships that could be construed as a potential conflict of interest.

## Publisher's Note

All claims expressed in this article are solely those of the authors and do not necessarily represent those of their affiliated organizations, or those of the publisher, the editors and the reviewers. Any product that may be evaluated in this article, or claim that may be made by its manufacturer, is not guaranteed or endorsed by the publisher.

## Supplementary Material

Supplementary Table S1 Least square means (lsmeans) comparison of drought tolerance indices based on grain yield of durum wheat study panel under FDS and FNS conditions combined from three seasons (2016–2018). Pedigree, GY_FNS: lsmeans of grain yield from FNS treatments at Holeta and Debre Zeit, GY_FDS, lsmeans of grain yield from FDS treatments at Dera and Melkassa, DSI, drought susceptibility index, RDI, relative drought index, STI, stress tolerance index, GMP, geometric mean productivity, TOL, tolerance index, and YSI, yield stability index. Drought indices values and their respective ranks were indicated. Yellow formatting indicates the top five selected accessions based on combined ranks of DSI, RDI, TOL, and YSI **(A)**, as well as based on combined ranks of STI and GMP **(B)**.

Supplementary Table S2 Marker-trait associations (MTAs) were detected at–log_10_*p* ≥ 4.0 for drought indices calculated from grain yield and traits that were significantly (*p* < 0.001) positively correlated with grain yield under FDS and FNS.

Supplementary Table S3 Significant (–log10*p* ≥ 4.0) MTAs grouped into QTLs for drought indices calculated from grain yield (GY) and traits that were significantly (*p* < 0.001) andpositively correlated with grain yield under FDS and FNS. The chromosome interval of the hotspots is indicated by a red rectangle.

Supplementary Figure S1 Scatter plot matrix showing the relationships among drought indices and grain yield (GY) from which drought indices were calculated. GY_FNS, grain yield lsmeans from FNS at Holeta and Debre Zeit; GY_FDS, grain yield lsmeans from FDS at Dera and Melkassa; DSI, drought susceptibility index; RDI, relative drought index; STI, stress tolerance index; GMP, geometric mean productivity; TOL, tolerance index; YSI, yield stability index.

Supplementary Figure S2 Manhattan plots of single-nucleotide polymorphism (SNP) marker-trait associations for drought susceptible index (DSI), geometric mean productivity (GMP), relative drought index (RDI), stress tolerance index (STI), tolerance index (TOL), and yield stability index (YSI) derived from grain yield (GY). The *x*-axis indicates 14 chromosomes from (left to right) and *y*-axis represents –log_10_*p* value. Marker-trait associations (MTAs) are significant at –log_10_*p* ≥ 6 (solid green line).

Supplementary Figure S3 Manhattan plots of single-nucleotide polymorphism (SNP) marker-trait associations for drought susceptible index (DSI), geometric mean productivity (GMP), relative drought index (RDI), stress tolerance index (STI), tolerance index (TOL), and yield stability index (YSI) derived from days to grain filling (DGF). The *x*-axis indicates 14 chromosomes from (left to right) and *y*-axis represents –log_10_*p* value. Marker-trait associations (MTAs) are significant at –log_10_*p* ≥ 6 (solid green line).

Supplementary Figure S4 Manhattan plots of single-nucleotide polymorphism (SNP) marker-trait associations for drought susceptible index (DSI), geometric mean productivity (GMP), relative drought index (RDI), stress tolerance index (STI), tolerance index (TOL), and yield stability index (YSI) calculated from SPAD. The *x*-axis indicates 14 chromosomes from left to right and *y*-axis represents –log_10_*p* value. Marker-trait associations (MTAs) are significant at –log_10_*p* ≥ 6 (solid green line).

Supplementary Figure S5 Manhattan plots of single-nucleotide polymorphism (SNP) marker-trait associations for drought susceptible index (DSI), geometric mean productivity (GMP), relative drought index (RDI), stress tolerance index (STI), tolerance index (TOL), and yield stability index (YSI) derived from seed per spike (SPS). The *x*-axis indicates 14 chromosomes from left to right and *y*-axis represents –log_10_*p* value. Marker-trait associations (MTAs) are significant at –log_10_*p* ≥ 6 (solid green line).

Supplementary Figure S6 Manhattan plots of single-nucleotide polymorphism (SNP) marker-trait associations for drought susceptible index (DSI), geometric mean productivity (GMP), relative drought index (RDI), stress tolerance index (STI), tolerance index (TOL), and yield stability index (YSI) derived from harvesting index (HI). The *x*-axis indicates 14 chromosomes from left to right and *y*-axis represents –log_10_*p* value. Marker-trait associations (MTAs) are significant at –log_10_*p* ≥ 6 (solid green line).

Supplementary Figure S7 Manhattan plots of single-nucleotide polymorphism (SNP) marker-trait associations for drought susceptible index (DSI), geometric mean productivity (GMP), relative drought index (RDI), stress tolerance index (STI), tolerance index (TOL), and yield stability index (YSI) calculated from thousand kernel weight (TKW). The *x*-axis indicates 14 chromosomes from left to right and *y*-axis represents –log_10_*p* value. Marker-trait associations (MTAs) are significant at –log_10_*p* ≥ 6 (solid green line).

## Abbreviations

DGF: Days to grain filling
DSI: Drought susceptibility index
EBI: Ethiopian Biodiversity Institute
EIAR: Ethiopian Institute of Agricultural Research
FDR: False discovery rate
FDS: Filed drought stress
FNS: Field non-stress
GMP: Geometric mean production
GWAS: Genome-wide association study
GY: Grain yield
HI: Harvest index
LD: Linkage disequilibrium
LOD: Logarithm of odds
Lsmeans: Least square means
MTA: Marker-trait analysis
P: Probability value
PCA: Principal component analysis
PVE: Phenotypic variance explained
QTLs: Quantitative trait loci
r: Pearson's correlation coefficient
RDI: Relative drought index
SNP: Single-nucleotide polymorphism
SPAD: Soil plant analysis development
SPS: Seeds per spike
STI: Stress tolerance index
TKW: Thousand kernel weight
TOL: Tolerance index
YSI: Yield stability index.
