# Supplementary figures and images for: Association Mapping of Drought Tolerance Indices in Ethiopian Durum Wheat (Triticum turgidum ssp. durum)
Source: Front Plant Sci. 2022 May 26;13:838088. doi: 10.3389/fpls.2022.838088 (PMC9178276; doi:10.3389/fpls.2022.838088)

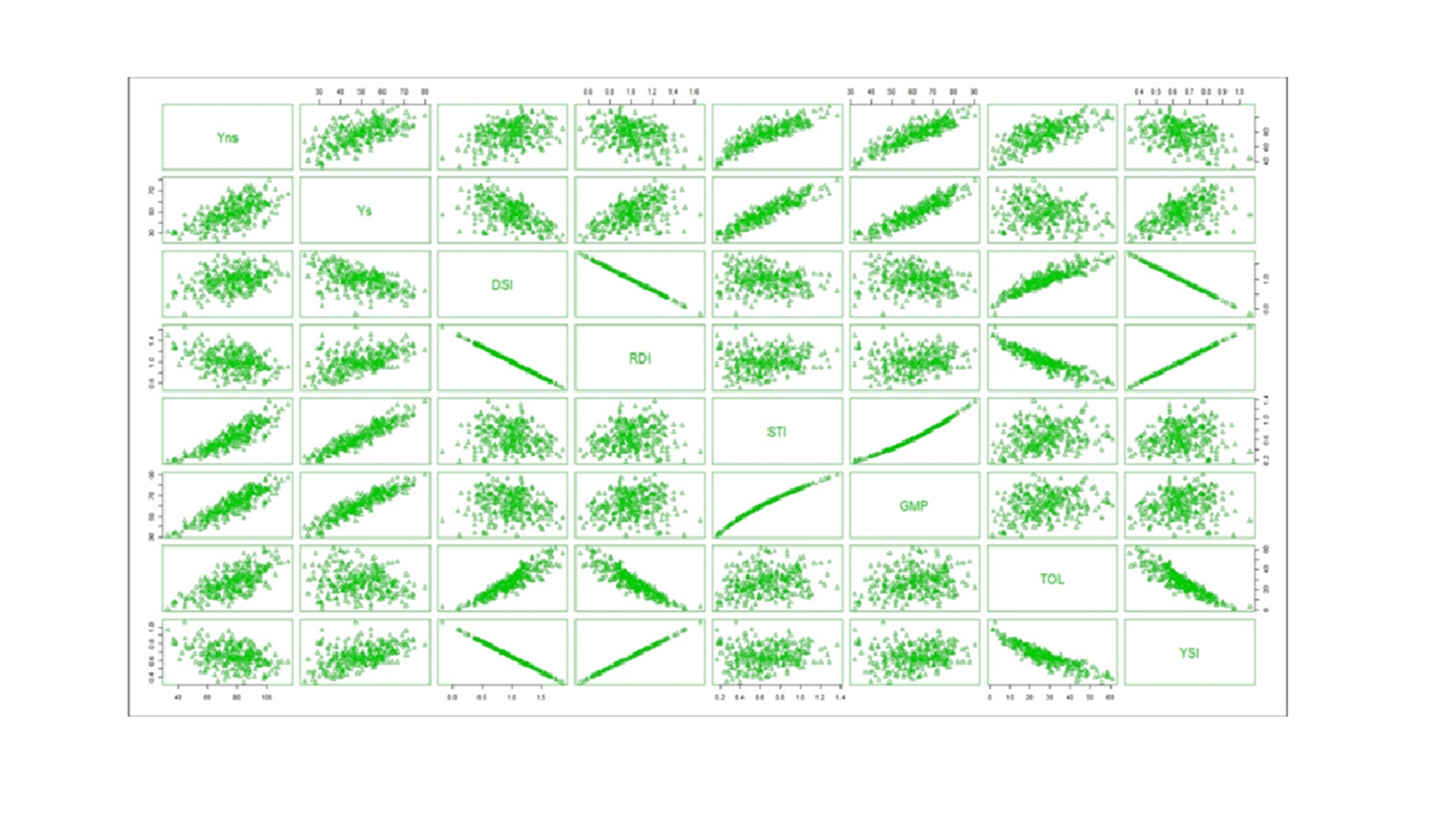

Supplement: Supplementary Figure S1 — Scatter plot matrix showing the relationships among drought indices and grain yield (GY) from which drought indices were calculated. GY_FNS, grain yield lsmeans from FNS at Holeta and Debre Zeit; GY_FDS, grain yield lsmeans from FDS at Dera and Melkassa; DSI, drought susceptibility index; RDI, relative drought index; STI, stress tolerance index; GMP, geometric mean productivity; TOL, tolerance index; YSI, yield stability index. [file Image_1.TIF]

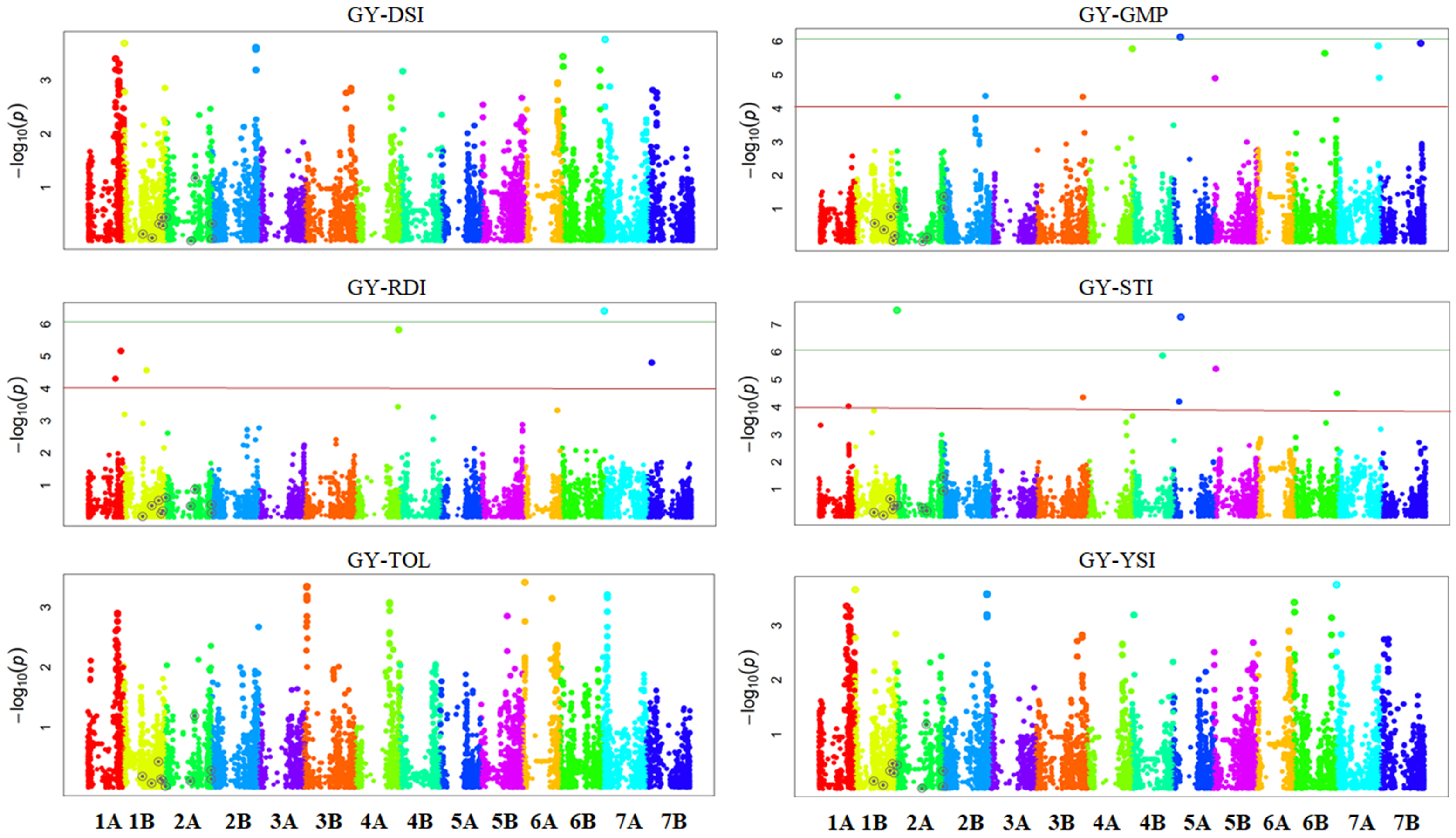

Supplement: Supplementary Figure S2 — Manhattan plots of single-nucleotide polymorphism (SNP) marker-trait associations for drought susceptible index (DSI), geometric mean productivity (GMP), relative drought index (RDI), stress tolerance index (STI), tolerance index (TOL), and yield stability index (YSI) derived from grain yield (GY). The x-axis indicates 14 chromosomes from (left to right) and y-axis represents –log10p value. Marker-trait associations (MTAs) are significant at –log10p ≥ 6 (solid green line). [file Image_2.TIF]

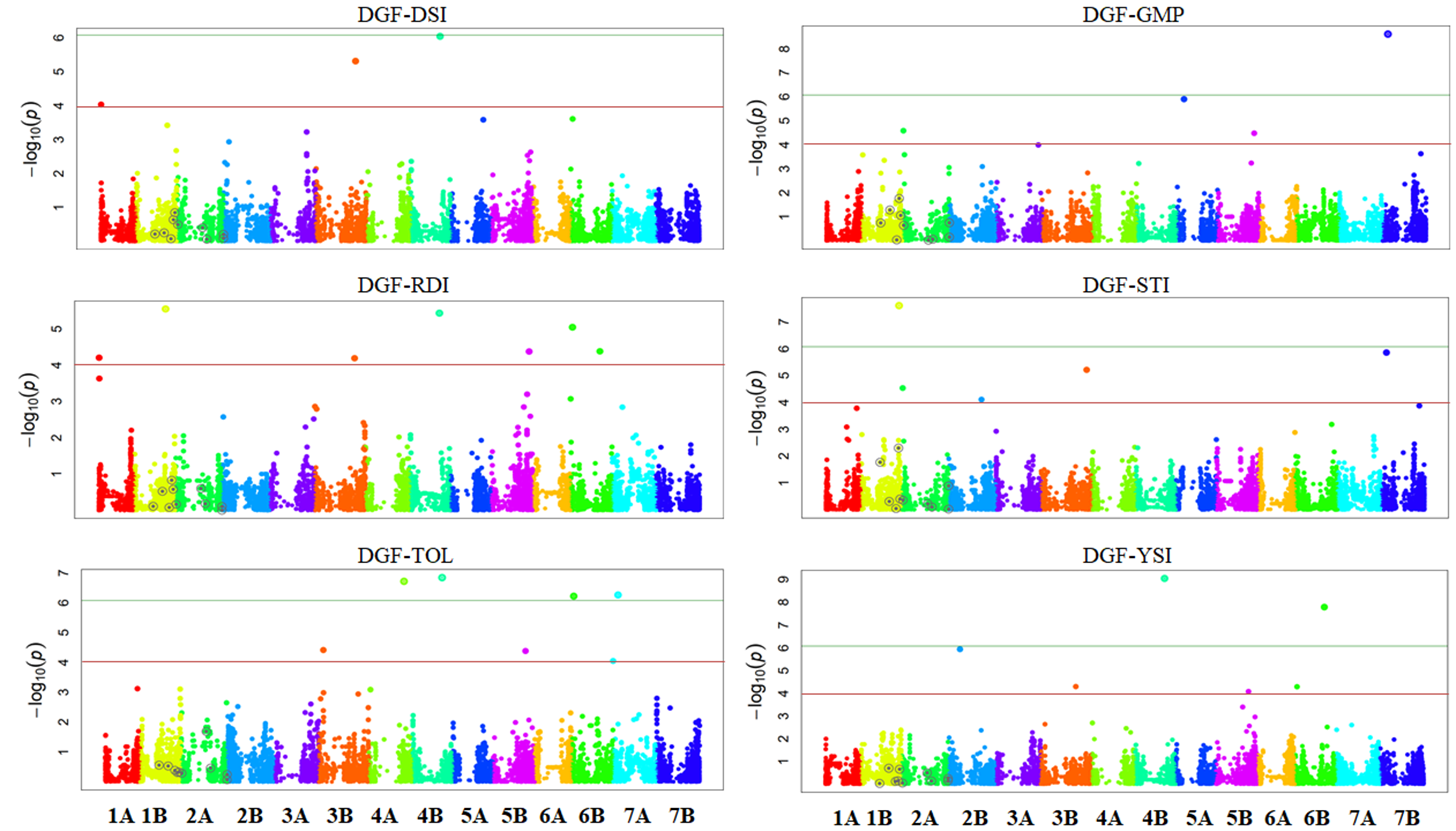

Supplement: Supplementary Figure S3 — Manhattan plots of single-nucleotide polymorphism (SNP) marker-trait associations for drought susceptible index (DSI), geometric mean productivity (GMP), relative drought index (RDI), stress tolerance index (STI), tolerance index (TOL), and yield stability index (YSI) derived from days to grain filling (DGF). The x-axis indicates 14 chromosomes from (left to right) and y-axis represents –log10p value. Marker-trait associations (MTAs) are significant at –log10p ≥ 6 (solid green line). [file Image_3.TIF]

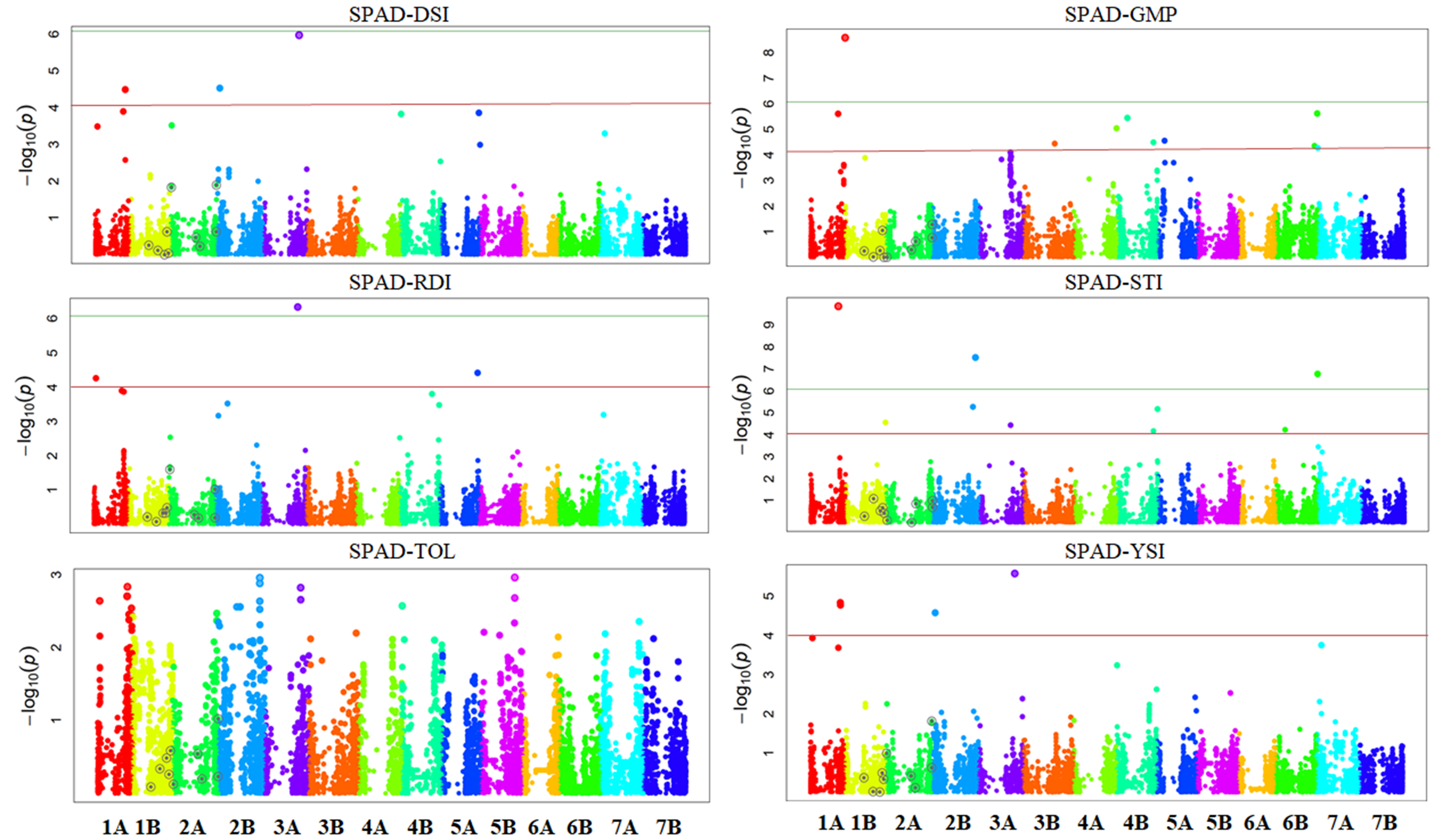

Supplement: Supplementary Figure S4 — Manhattan plots of single-nucleotide polymorphism (SNP) marker-trait associations for drought susceptible index (DSI), geometric mean productivity (GMP), relative drought index (RDI), stress tolerance index (STI), tolerance index (TOL), and yield stability index (YSI) calculated from SPAD. The x-axis indicates 14 chromosomes from left to right and y-axis represents –log10p value. Marker-trait associations (MTAs) are significant at –log10p ≥ 6 (solid green line). [file Image_4.TIF]

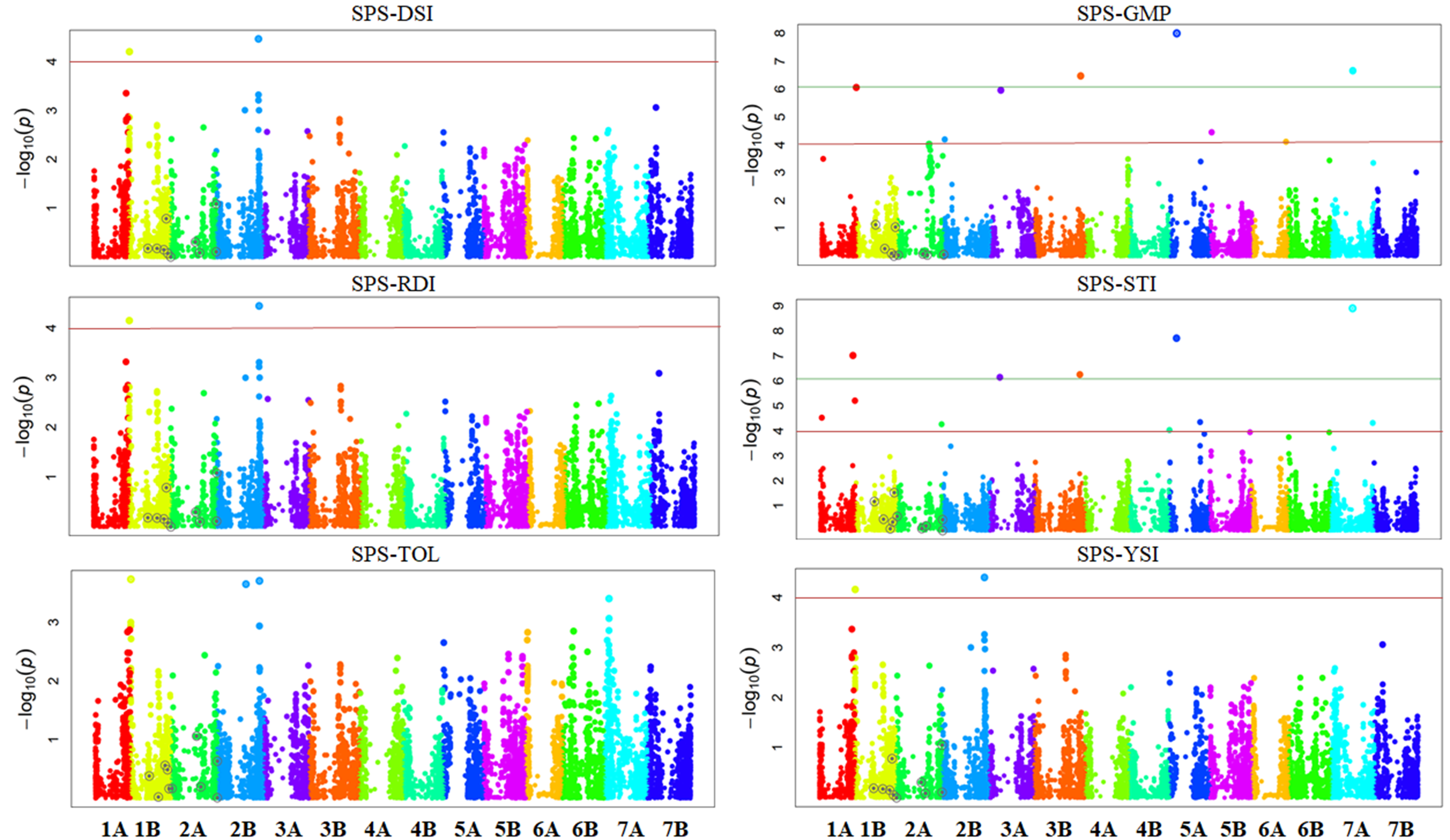

Supplement: Supplementary Figure S5 — Manhattan plots of single-nucleotide polymorphism (SNP) marker-trait associations for drought susceptible index (DSI), geometric mean productivity (GMP), relative drought index (RDI), stress tolerance index (STI), tolerance index (TOL), and yield stability index (YSI) derived from seed per spike (SPS). The x-axis indicates 14 chromosomes from left to right and y-axis represents –log10p value. Marker-trait associations (MTAs) are significant at –log10p ≥ 6 (solid green line). [file Image_5.TIF]

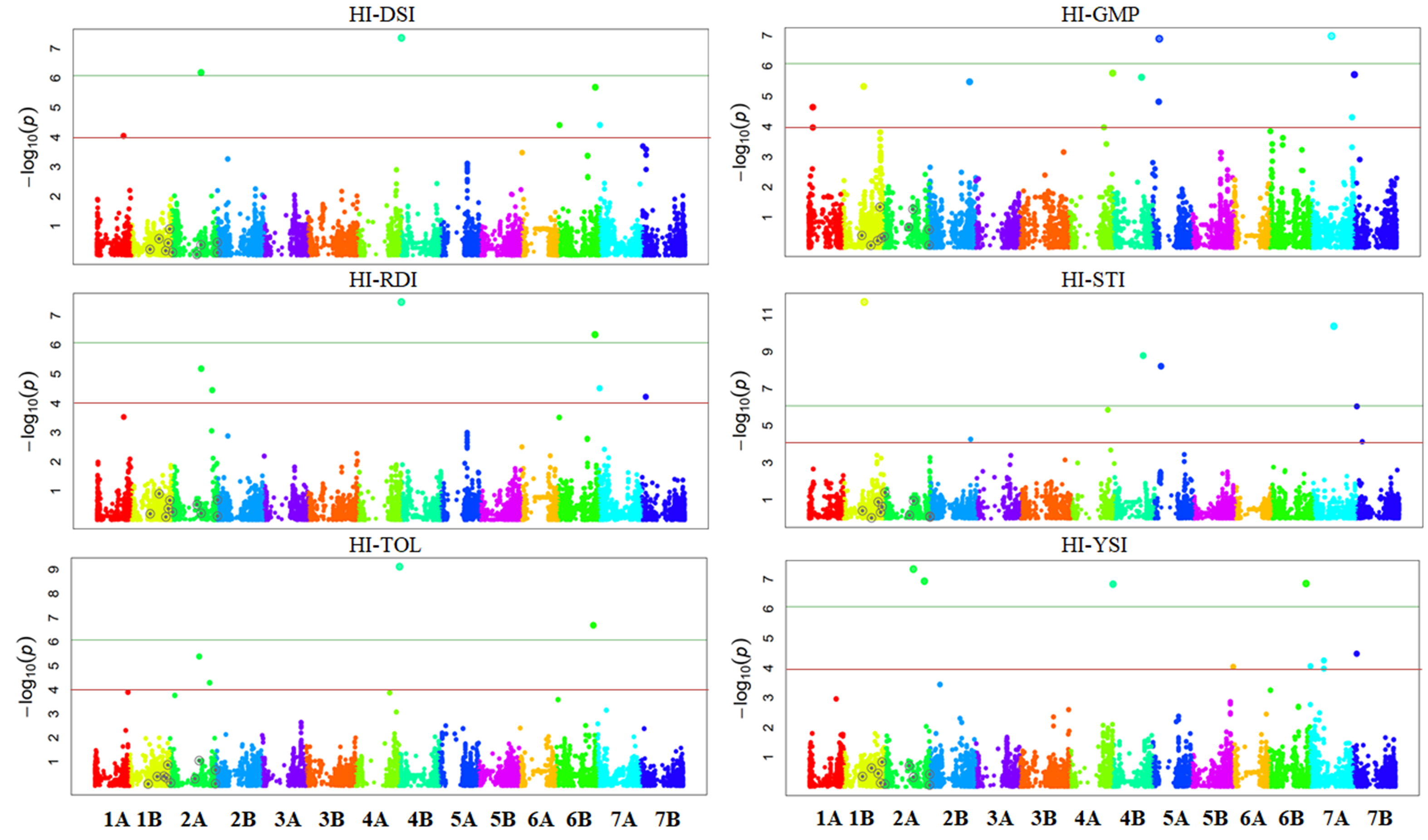

Supplement: Supplementary Figure S6 — Manhattan plots of single-nucleotide polymorphism (SNP) marker-trait associations for drought susceptible index (DSI), geometric mean productivity (GMP), relative drought index (RDI), stress tolerance index (STI), tolerance index (TOL), and yield stability index (YSI) derived from harvesting index (HI). The x-axis indicates 14 chromosomes from left to right and y-axis represents –log10p value. Marker-trait associations (MTAs) are significant at –log10p ≥ 6 (solid green line). [file Image_6.TIF]

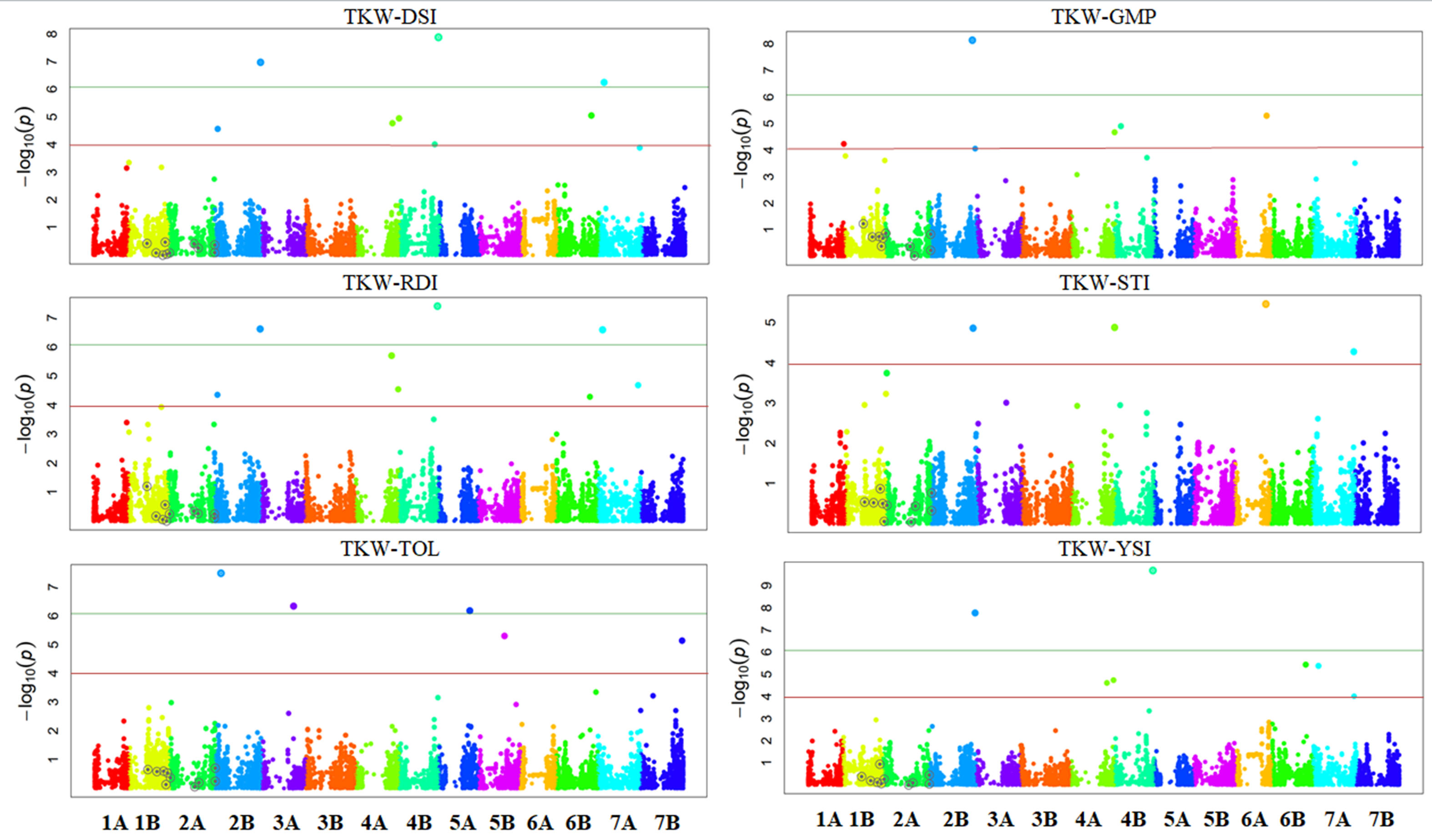

Supplement: Supplementary Figure S7 — Manhattan plots of single-nucleotide polymorphism (SNP) marker-trait associations for drought susceptible index (DSI), geometric mean productivity (GMP), relative drought index (RDI), stress tolerance index (STI), tolerance index (TOL), and yield stability index (YSI) calculated from thousand kernel weight (TKW). The x-axis indicates 14 chromosomes from left to right and y-axis represents –log10p value. Marker-trait associations (MTAs) are significant at –log10p ≥ 6 (solid green line). [file Image_7.TIF]
